# Empirical dynamic modeling of the association between ambient PM_2.5_ and under-five mortality across 2851 counties in Mainland China, 1999–2012

**DOI:** 10.1016/j.ecoenv.2022.113513

**Published:** 2022-06-01

**Authors:** Sameh M.M. Alnwisi, Chengwei Chai, Bipin Kumar Acharya, Aaron M. Qian, Shiyu Zhang, Zilong Zhang, Michael G. Vaughn, Hong Xian, Qinzhou Wang, Hualiang Lin

**Affiliations:** a Department of Epidemiology, School of Public Health, Sun Yat-sen University, Guangzhou, China; b Guangzhou Women and Children's Medical Center, Guangzhou Medical University, Guangzhou 510623, China; c Department of Psychology, College of Arts and Sciences Saint Louis University, 3700 Lindell Boulevard, Saint Louis, MO 63108, USA; d School of Social Work, College for Public Health & Social Justice, Saint Louis University, Tegeler Hall, 3550 Lindell Boulevard, Saint Louis, MO 63103, USA; e Department of Epidemiology and Biostatistics, College for Public Health & Social Justice, Saint Louis University, 3545 Lafayette Avenue, Saint Louis, MO 63104, USA; f Research Institute of Neuromuscular and Neurodegenerative Diseases and Department of Neurology, Qilu Hospital, Cheeloo College of Medicine, Shandong University, Jinan, China

**Keywords:** Ambient air pollution, Fine particulate matter pollution, Under five mortality, Empirical dynamic model

## Abstract

**Background:**

Ambient fine particulate matter (PM_2.5_) pollution has been associated with mortality from various diseases, however, its association with under-five mortality rate (U5MR) has remained largely unknown.

**Methods:**

Based on the U5MR data across 2851 counties in Mainland China from 1999 to 2012, we employed approximate Bayesian latent Gaussian models to assess the association between ambient PM_2.5_ and U5MR at the county level for the whole nation and sub-regions. GDP growth rate, normalized difference vegetation index (NDVI), temperature, and night-time light were included as covariates using a smoothing function. We further implemented an empirical dynamic model (EDM) to explore the potential causal relationship between PM_2.5_ and U5MR.

**Results:**

We observed a declining trend in U5MR in most counties throughout the study period. Spatial heterogeneity in U5MR was observed. Nationwide analysis suggested that each 10 µg/m^3^ increase in annual concentration of PM_2.5_ was associated with an increase of 1.2 (95% CI: 1.0 – 1.3) per 1000 live births in U5MR. Regional analyses showed that the strongest positive association was located in the Northeastern part of China [1.8 (95% CI: 1.4 – 2.1)]. The EDM showed a significant causal association between PM_2.5_ and U5MR, with an embedding dimension of 5 and 7, and nonlinear values θ of 4 and 6, respectively.

**Conclusion:**

China exhibited a downward trend in U5MR from 1999 to 2012, with spatial heterogeneity observed across the country. Our analysis reveals a positive association between PM_2.5_ and U5MR, which may support a causal relationship.

## Highlights

• Ambient PM_2.5_ was associated with an increase of 0.11 per 1000 live births in U5MR.
• Empirical dynamic model suggests a causal association between PM_2.5_ and U5MR.
• Bayesian spatiotemporal interaction model is well-fitted to estimate ecological factors impact on mortality.

## 1 Introduction

Defined as the probability of dying between birth and five years of age and expressed as per 1000 live births (Song et al., 2016), under-five mortality rate (U5MR) is a key indicator used to scrutinize child survival and environmental and economic status (Gortmaker and Wise, 1997). Despite substantial efforts to reduce child mortality across the globe, it is estimated that there are about 16,000 deaths among children every day (Corsi and Subramanian, 2014, You et al., 2010). The United Nations Millennium Development Goals (MDGs), signed in September 2000, aimed to reduce U5MR by two-thirds by 2015 (Gaffey et al., 2015), however, only a few countries have achieved this goal (Wang et al., 2014). MDGs have more recently been replaced by the newly launched Sustainable Development Goals (SDGs) to end preventable deaths among children under five years of age by 2030 (Van de Pas et al., 2017).

Coinciding with the rapid development of civilization and the economy in recent decades, U5MR has exhibited a dramatic decrease in China. In 2008, the U5MR decreased by two-thirds compared to the rate in 1990 (Wang et al., 2014, Feng et al., 2012). Despite the overall decrease in U5MR, an elevated rate still exists in the Northern and Southwestern regions compared to the rest of the nation. This disparity may be partially explained by high geographical heterogeneity (Wang et al., 2016). To achieve the goal of the SDGs, it is important to investigate the risk factors associated with mortality risk among children. Several studies have previously investigated the relationship between U5MR and various potential risk factors. Malnutrition, lifestyle, healthcare, and various socioeconomic factors have been identified as crucial determinants of U5MR (Van Malderen et al., 2019, Davenport et al., 2017, De Neve et al., 2020).

In addition to the aforementioned crucial determinants, ambient particulate matter pollution has been identified as a potential preventable risk factor of various health outcomes (Yu, 2020, Zhang et al., 2020, He et al., 2022). For instance, one study by Anwar investigated the impact of PM_2.5_ on child mortality, and found that a single unit increase in PM_2.5_ leads to an increase of 14.5 in U5MR (Anwar et al., 2021). Yitzhaks et al. estimated that each 3 µg/m^3^ increase in ambient PM_2.5_ was associated with an increase of 4.4% in all-cause mortality (Yitshak-Sade et al., 2019). Woodruff et al. reported that postnatal deaths due to respiratory diseases were significantly associated with ambient PM_2.5_ exposure (Woodruff et al., 2006). Another study by Dockery et al. observed a strong association between air pollution and mortality due to lung cancer and pulmonary diseases (Dockery et al., 1993). Other studies have also demonstrated significant adverse effects in children due to air pollution even at low levels (Rice et al., 2016, Janke, 2014) Exposure to ambient air pollution during pregnancy has also been reported to be associated with the adverse pregnancy outcomes of the newborns (Pejhan et al., 2019, Liang et al., 2019). Taken together, it is reasonable to hypothesize that ambient PM_2.5_ exposure may contribute to under-five mortality.

Nighttime Light (NTL) data can numerically characterize the intensity of urbanization, socioeconomic, artificial light, and human activities (Li et al., 2020). Several studies have used NTL data to assess the association between urbanization, poverty, and mortality (Ma et al., 2012, Noor et al., 2008, Wang et al., 2012). Furthermore, nighttime light data have been used in predicting PM_2.5_ concentrations (Li et al., 2017). Normalized Difference Vegetation Index (NDVI) is one of the most popular remotely vegetation indices and a valuable way to understand vegetation health, environmental change, and air pollution concentrations (Tucker et al., 2005). Researchers are increasingly exploring the effect of vegetation on health outcomes, including survival and mortality rates (Wilker et al., 2014, Hystad et al., 2014). Both were introduced as confounding covariates.

Ecological regression models have been widely used to assess the association between mortality and risk factors. Although several studies have examined the association between air pollution and child health, most have not focused on asserting causal relationships (Yu et al., 2020). Therefore, we aim to conduct a nation-wide study to investigate the association between ambient PM_2.5_ and U5MR across 2851 Chinese counties from1999 to 2012. Additionally, we seek to explore its effect on subnational regions and investigate potential causality.

## 2 Materials and methods

### 2.1 Data source

We used annual U5MR data (expressed by number of deaths per 1000 live births) across 2851 counties of China over the period of 1996–2012. Mortality estimation and spatiotemporal models were used to synthesize the data from several surveys, surveillance systems, censuses, as well as the Annual Report System on Maternal and Child Health (ARMCH) (Wang et al., 2016). To address stochastic variability in deaths in addition to mortality due to natural causes, Gaussian process regression was used to predict the U5MR (Wang et al., 2016). The U5MR dataset was the first to measure the trends and levels of child mortality in the 2851 Chinese counties from 1996–2012. In this study, data from 1999 to 2012 was used according to the availability of ambient PM_2.5_ data and other covariates.

Yearly data for ambient fine particulate matter (PM_2.5_) from 1999 to 2012 was obtained from the United States NASA’s socioeconomic data and application center (SADAC) (Socioeconomic Data and Applications Center | SEDAC (columbia.edu). Global annual PM_2.5_ gridded data consisted of annual concentrations of ground-level PM_2.5_, with dust and sea-salt removed at a geographical resolution of 0.01°* 0.01° (approximately 1 km * 1 km). These data combine Aerosol Optical Depth (AOD) retrievals from multiple satellites, including the NASA Moderate Resolution Imaging Spectroradiometer (MODIS), Multi-angle Imaging Spectro Radiometer (MISR), and the Sea-Viewing Wide Field-of-View Sensor (SeaWiFS). According to Hammer, van Donkelaar et al. PM_2.5_ estimates are highly consistent with globally distributed ground monitors from the WHO Global Ambient Air Quality Database (Organization, 2018), with a cross-validation value of R^2^ = 0.81. Moreover; Geographically weighted regression is applied to account for PM_2.5_ residual with ground monitors yielding a cross-validation value of R^2^ = 0.90 (Hammer et al., 2020). The data are distributed as Geotiff files and were extracted using a mask tool at the county level. The annual values were summarized by zonal statistics tools using ArcMap 10.6.

Annual night-time light data enables investigators to monitor human activities, economic status, estimating exposed population size, and the impact of light pollution. Data was ascertained from the Defense Meteorological Satellite Program DMSP/Operational Linescan System OLS. We downloaded the intercalibrated stable DMSP/OLS NTL (version 4) from the earth observation group (https://eogdata.mines.edu/dmsp/downloadV4composites.html), with a spatial resolution of 30 arc-seconds, spinning coverage of − 180–180° in longitude and − 65–75° in latitude. The NTL stable version values range from 0 to 63, with zero background noise.

Generated by NASA tools, the Normalized Difference Vegetation Index (NDVI) provides constant, long-term data on global surface vegetation based on remotely sensed observations (https://giovanni.gsfc.nasa.gov/). It is computed by dividing the difference between the reflectance measurement of the near-infrared band and the red band by their sum. This method uses 0.05-degree Climate Modeling Grid CMG spatial resolution of 5,600-meter (m) pixels and monthly temporal resolution. Terra MODIS NDVI data supplied by the MOD13C2 version 6 at per pixel basis, provided 16-day composite reflectance data, angular information, and spatial statistics such as mean, standard deviation, with a value ranging between − 1 and + 1. Data were collected for January 1999 to December 2012.

Gridded temperature data were collected from the Copernicus Atmosphere Monitoring Service (CAMS reanalysis-ERA5) (https://cds.climate.copernicus.eu/). It utilized vertical coverage 2 m above the sea surface and land, and vertical resolution of 4 levels of the European Centre for Medium-Range Weather Forecasts ECMWF surface model. The data have been gridded to a regular latitude-longitude grid with 0.01°* 0.01° horizontal resolution degrees. In the present study, monthly temperature data were collected from January 1999 to December 2012 with 52 missing counties.

Gross domestic product (GDP) data at the county level were collected from the Chinese Statistical Yearbook for the corresponding years from the National Bureau of Statistics (www.stats.gov.cn). Data on GDP was missing for 453 counties.

Missing values were estimated by implementing the Inverse Distance Weighting (IDW) interpolation method, ArcMap 10.6.

### 2.2 Statistical analysis

The present study included data from 2851 Chinese counties across 31 provincial regions from 1999 to 2012. The health effects of ambient PM_2.5_ on U5MR were estimated at the national level and in six subnational regions based on administrative areas in China (East and North China, Northeast, and Northwest China, Southwest, and Southcentral China). Fig. S1 shows the six regions. In the analysis, the annual GDP growth rate, NDVI, temperature, and night-time light data were included as smooth random-effect covariates (Gómez-Rubio, 2020).

This study is based on longitudinal data design, implementing INLA models in hierarchal framework. Bayesian hierarchical models (Lawson, 2018) apply more than one coefficient to compute the regression model, thus it is suitable for parameter estimation, especially for large and decentralized areas (Spiegelhalter et al., 2002). Moreover, it also introduces the simplest way to account for structured stochastic (latent) variability in the data through distributed independent and identical random effects, which account for spatial and temporal autocorrelation, and increases the Integrated Nested Laplace Approximation (INLA) efficiency (Gómez-Rubio, 2020, Illian et al., 2013). We applied three competing models (classic, nonparametric, and spatiotemporal interaction) with different space-time formulas, (1), (2), (3) (Blangiardo et al., 2013), to explore the spatial and temporal distribution of U5MR and to evaluate the impact of PM_2.5_ on U5MR. According to the Deviance Information Criterion (DIC) and Watanabe-Akaike Information Criterion (WAIC) values, we selected the best-fitted model (Spiegelhalter et al., 2002). We also calculated the Mean Square Error (MSE) and Mean Absolute Error (MAE) to enhance the criterion selection of the best fitted model. Importantly, we used the empirical dynamic model (EDM) (Ye et al., 2018) to estimate potential causality between U5MR (the response variable) and its potential driver, ambient PM_2.5_, by employing the convergence cross mapping (CCM) function (Ye et al., 2018, Chang et al., 2017).

### 2.3 Bayesian spatiotemporal model

Bayesian modeling relies on the ability to calibrate posterior distributions. By providing observed data and prior distribution information, estimates for all the related model parameters can be made. The prior distribution is the distribution of the parameter prior to any observed data. However, the posterior distribution is the distribution of the parameter, following given data, which is the crucial part of Bayesian inference.

To assess the spatiotemporal trend of U5MR and explore the influence of ambient PM_2.5_ at the national and sub-national levels, we implemented Bayesian inference latent Gaussian distribution within the Integrated Nested Laplace Approximation (INLA) (Team, 2013). Gaussian likelihood can be expressed using the formula:

$$y_{\mathrm{i}t}\sim \;\mathrm{Normal}\left(\eta_{\mathrm{it}},\;\sigma_{e}^{2}\right)$$

where$\;y_{\mathrm{i}t}$ represents the rate of U5MR for the *i-th* county area (*i* = 1, 2, …., 2851), in given year*t* (*t* *=* 1999, 2000…2012), $\sigma_{e}^{2}$ is the measurement of error variance, and $\eta_{\mathrm{it}}$ is the linear predictor model of INLA for $y_{\mathrm{i}t}$.

**Model 1:** The classic parametric formulation has been introduced previously (Bernardinelli et al., 1995). This analysis can estimate the effect of a set of factors on response covariates in a regression model, and account for spatial and temporal correlation. Thus, the first linear predictor model can be assumed as follows:

(1) $$\begin{array}{c} \eta_{\mathrm{it}}=\alpha +\beta_{\mathrm{PM}\;}x_{\mathrm{PM},\mathrm{i}}+f\left(\beta_{\mathrm{GDP}\;}+\;x_{\mathrm{GDP},\mathrm{i}}\right)\\ \;\;\;\;\;\;\;\;\;\;\;+f\left(\beta_{\mathrm{NDVI}\;}+\;x_{\mathrm{NDVI},\mathrm{i}}\right)+f\left(\beta_{\mathrm{NTL}\;}+\;x_{\mathrm{NTL},\mathrm{i}}\right)\\ \;\;\;\;\;\;\;\;\;\;\;+f\left(\beta_{\mathrm{TEMP}\;}+\;x_{\mathrm{TEMP},\mathrm{i}}\right)+v_{i}+\vartheta_{i}+\left(\beta_{0\;}+\delta_{i}\right)t \end{array}$$

*The f(.)* function is used to specify random effects (spatial, temporal, and covariates) with different latent models’ specifications. In our study, it includes an influencing covariate (NDVI, TEMP, GDP, and NTL), and an index to map the effect on the observation (ID. area) referring to county ID and model type specification. The formula can be written as:

*f (Covariate, ID·area, model="…")*

where α is the intercept of the overall U5MR in all the 2851 counties (by default α was assigned Gaussian prior with mean and precision equal to 0); $\beta_{\mathrm{PM}}$ is the fixed effect coefficient of covariate PM_2.5_ ($x_{\mathrm{PM},\mathrm{i}}$), for *i-th* county area, assuming default Gaussian prior,$\;\beta_{\mathrm{PM}}\sim N\left(0,\;0.01\right)$, Blangiardo et al. (2013) with zero mean and precision equal to 0.01; and the spatially varying coefficients for the smooth random-effect covariates: GDP growth rate ${(x}_{\mathrm{GDP}})$, NDVI$\;{(x}_{\mathrm{NDVI}})$, night-time light data$\;{(x}_{\mathrm{NTL}})$, and temperature ${(x}_{\mathrm{TEMP}})$. By default, all assuming Gaussian distribution with zero mean and precision $\sigma_{\beta }^{2}\;$,$\;\left\{\beta_{\mathrm{GDP},\mathrm{i}},\beta_{\mathrm{NDVI},\mathrm{i}},\beta_{\mathrm{NL},\mathrm{i}},\beta_{\mathrm{TEMP},\mathrm{i}}\right\}\sim N(0,\sigma_{\beta }^{2}$), where $\sigma_{\beta }^{2}\;$is assigned Gamma priors with parameters $\sigma_{\beta }^{2}\sim \mathrm{gamma}\left(1,\;0.0005\right)$. (Pusponegoro, 2018, Schrödle and Held, 2011) The term $\xi_{i}=v_{i}+\vartheta_{i}\;$represents the overall spatial unstructured residual $\;v_{i}$ and structured residual $\vartheta_{i}$ components, assuming Besag-York-Mollie (BYM) specifications (Besag et al., 1991). The unstructured random effect $v_{i}$, is constructed to model variation of spatial residuals not modeled by structure random effects, and $v_{i}$ was assigned Normal prior, where $v_{i}\sim N\left(0,\sigma_{v}^{2}\right),$ in which the log-precision $\sigma_{v}$ was assigned $\sigma_{v}\sim$log-gamma (1, 0.0005) by default. However, the structured$\vartheta_{i}$￼, which is used to capture the spatial autocorrelation, were modeled via the Besag Conditional Auto-Regressive CAR, where $\vartheta_{i}\;$￼is normally distributed with a mean equal to the average value of its neighbors and has unknown variance:

$$\vartheta_{i}|\vartheta_{j},\sigma_{v}\left(\frac{1}{\sum_{j=1}^{m}\omega_{\mathrm{ij}}}\;\sum_{j\in \delta_{i}}\omega_{\mathrm{ij}}\vartheta_{j},\;\frac{1}{n_{\delta_{i}}\sigma_{\vartheta }}\right)\mathrm{i}\neq j$$

where the mean of each $\vartheta_{i}$ is defined as a weighted average of $\vartheta_{j}$, and $n_{\delta_{i}}$ is the number of neighbors. The spatial weight $\omega_{\mathrm{ij}}$ = 1 if ⅈ and j are adjacent neighbors or 0 if they are not (Kandhasamy and Ghosh, 2017). The conditional precision parameter$\;\sigma_{\vartheta }$ was assigned Gamma ~ (1, 0.0005). $\beta_{0}$ represents the global trend effect, and $\delta_{i}$ describes the differences between $\beta_{0}$ and area-specific trend, assuming Gaussian prior specification, whereas $\delta_{i}\sim N\left(0,\sigma_{\delta }^{2}\right)\;$, in which the precision assigned $\sigma_{\delta }^{2}\sim \mathrm{gamma}\left(1,\;0.0005\right)$.whereas tis time variable.

**Model 2:** The second linear predictor model, using dynamic non-parametric formulation, can be described as follows (Knorr-Held, 2000):

(2) $$\begin{array}{c} \;\eta_{\mathrm{it}}=\alpha +\beta_{\mathrm{PM}\;}x_{\mathrm{PM},\mathrm{i}}+f\left(\beta_{\mathrm{GDP}\;}+\;x_{\mathrm{GDP},\mathrm{i}}\right)\\ \;\;\;\;\;\;\;\;\;\;\;\;\;\;\;+f\left(\beta_{NDVI\;}+\;x_{\mathrm{NDVI},\mathrm{i}}\right)+\;f\left(\beta_{\mathrm{NL}\;}+\;x_{\mathrm{NL},\mathrm{i}}\right)\\ \;\;\;\;\;\;\;\;\;\;\;\;\;\;\;+f\left(\beta_{\mathrm{TEMP}\;}+\;x_{\mathrm{TEMP},\mathrm{i}}\right)+v_{i}+\vartheta_{i}+\gamma_{t}+\varphi_{t} \end{array}$$

where $\xi_{i}=\vartheta_{i}+v_{i}$, similar to formula (1), represents the overall spatial structured and unstructured components. The term $\omega =\gamma_{t}+\varphi_{t}$, represents the overall temporal unstructured${(\gamma }_{t}$) and structured$(\varphi_{t}$) effects. The uncorrelated or unstructured random effect $\gamma_{t}$, is used to explore time independent effects, assuming Gaussian prior $\gamma_{t}\sim N\left(0,\sigma_{\gamma }^{2}\right),$ with mean equal 0 and precision $\sigma_{\gamma }^{2}\sim \mathrm{gamma}\left(1,\;0.0005\right)$. By contrast, the structured temporal component $\varphi_{t}$, accounts for time dependent effects, presuming that the value for a county in a specific year is influenced by the value observed for that county in the previous year combined with a residual.

$\varphi_{t}$ was modeled via the first Random Walk process (RW1), specified as $\varphi_{t}=\varphi_{t-1}+∆\varphi$, where $∆\varphi =N(0,\sigma_{\varphi }^{2}$), and the conditional precision was assigned $\sigma_{\varphi }^{2}\sim \mathrm{gamma}\left(1,\;0.0005\right)$.

**Model 3:** To allow for interaction between space and time, we introduced δ_it_ parameter to formula 2 (Knorr-Held, 2000)_._ The resulting notation is:

(3) $$\begin{array}{c} \eta_{it}=\alpha +\beta_{PM\;}x_{PM,\mathrm{i}}+f\left(\beta_{GDP\;}+\;x_{GDP,\mathrm{i}}\right)\\ \;\;\;\;\;\;\;\;\;\;\;+f\left(\beta_{NDVI\;}+\;x_{NDVI,\mathrm{i}}\right)+f\left(\beta_{\mathrm{NL}\;}+\;x_{\mathrm{NL},\mathrm{i}}\right)\\ \;\;\;\;\;\;\;\;\;\;\;+f\left(\beta_{\mathrm{TEMP}\;}+\;x_{\mathrm{TEMP},\mathrm{i}}\right)+v_{i}+\vartheta_{i}+\gamma_{t}+\varphi_{t}+\delta_{\mathrm{it}} \end{array}$$

where $\delta_{\mathrm{it}}$ defines the unstructured spatiotemporal interaction, assuming no interaction between structured components; therefore, $\delta_{\mathrm{it}}$ checks for any residual spatiotemporal variation that is not explored by the spatial or temporal effects. Assuming Gaussian prior specification $\delta_{\mathrm{it}}\sim N(0,\sigma_{\delta }^{2}$), the precision of spatiotemporal random effect was assigned $\sigma_{\delta }^{2}\sim \mathrm{gamma}\left(1,\;0.0005\right)$. The estimated parameters are $\theta =\left\{\alpha ,\;\beta_{\mathrm{PM}},\;\vartheta ,\upsilon ,\gamma ,\varphi ,\delta \right\}$. The hyperparameter prior$\;\Psi =\left\{\tau \nu ,\tau \upsilon ,\tau \gamma ,\tau \varphi ,\tau \delta ,\tau \beta_{\mathrm{GDP}},\ldots ,\tau \beta_{\mathrm{TEMP}}\right\}$.

The prior distribution in the three models was assumed as INLA default specifications (Gómez-Rubio, 2020). The insertion of independent Gaussian i.i.d and conditional autoregressive CAR processes are adequate to compute uncertainties in small area variance, especially if geographic information of neighborhoods is provided. These inclusions are also suitable to account for unmeasured risk factors (Datta and Mandal, 2015).

We used the DIC and WAIC criteria to assess the performance of the models and selected the best-fitted model with the smallest DIC and WAIC, taking into consideration model complexity. We also calibrated the Mean Square Error (MSE) and Mean Absolute Error (MAE), to evaluate the predictive performance of the models as well. Like the DIC and WAIC, the lower the MSE and MAE values, the better the predicative performance of the model. The Bayesian evaluation was employed on R version 4.0.3 (R Core Team, Vienna, Austria) using the “INLA” package (www.r-inla.org) (Team, 2013, Rue et al., 2009).

### 2.4 Empirical dynamic model (EDM)

Additionally, an empirical dynamic model was conducted to explore the causality of the PM_2.5_ - U5MR associations, using the “EDM” package (0.7.5). Time-series data were normalized to improve data integrity and to avoid attractor distortion. EDM should be applied in a deterministic dynamic system due to its sensitivity to stochasticity. Although EDM allows for some stochastic covariates, the dynamic system as a whole should not be stochastic (Cummins et al., 2015). EDM constructs an attractor system in space from univariate or multivariate time-series data known as State Space Reconstruction (SSR), to assess the system characteristics and predict its dynamics (Sugihara, 1994, Sugihara and May, 1990). We used simplex projection to measure univariable deterministic dynamic embedding dimension and S-map to test nonlinearity.

#### 2.4.1 Convergent cross mapping

To identify the potential causal association between ambient PM_2.5_ and U5MR, we used a convergent cross mapping (CCM) approach within EDM. According to “Taken Theorem” testing, if the two variables observed from the same dynamic system are crucial, one should cross predict between the two variables (Sugihara et al., 2012, Takens, 1981). In other words, if PM_2.5_ causes U5MR, the characteristics and structural properties of PM_2.5_ will be traced in the U5MR dynamic system. In this case, the cross prediction of PM_2.5_ values from U5MR will improve, which is crucial to infer causality. However, since PM_2.5_ is independent of U5MR, and U5MR logically does not cause PM_2.5_, the cross prediction of U5MR from PM_2.5_ will fail. The crucial criterion for existing causality between two variables is to check the cross-mapping value ρ (Pearson correlation coefficient). If ρ is positive and gradually increases until convergence (a flat line), which corresponds with data length, then causality is significant. It should be noted that CCM estimates prediction skill in both directions (i.e., bidirectional).

#### 2.4.2 Null model (seasonal surrogate test)

To control the impact of seasonality and reject the null hypothesis that causality between variables is linked to shared seasonal fluctuation, we used two null model methods. The first is the “Ebisuzaki method” by generating randomized phases of Fourier transformed surrogate data and preserving any seasonal correlation. The second is the “Seasonal method” by creating average seasonal trend surrogate data. The null distribution ρ value from the two models were compared with the original times-series cross mapping ρ value (Ebisuzaki, 1997).

### 2.5 Sensitivity analysis

A Bayesian model is considered sensitive and non-robust when its posterior distribution changes dramatically with respect to the slight alteration of the prior distribution value. Model sensitivity to the specification of the prior parameter setting is a crucial part of the Bayesian sensitivity analysis, thus inappropriate prior specification may lead to inaccurate findings (Berger et al., 2000). Two sensitivity analysis approaches have been proposed, including global and local methods. In this study, we applied a local sensitivity approach to assess the change of the posterior value with respect to the prior parameter value. Sensitivity analyses were performed on model 3 as the best fitted model. Assuming different prior parameter specifications, the method can be expressed as follows:

**log-Gamma prior (default)** Defined with shape α and rate β parameters, where α = 1 and β = 0.00005.

**Penalized Complexity (PC) prior** Defined using probability statements on the model parameters; the PC prior for the precision τ is defined on the standard deviation σ=τ-1/2. PC priors have a single parameter which controls the amount of flexibility allowed in the model, u. Where u= 5 and ∝ is the probability of the event, so that $p\left(\sigma >\;u\right)=\;\propto$.

**Uniform prior** Defined with the notation$\;\left(\mu \right)\propto 1$; where $\log\left(\mu \right)=0-\log\left(2\right)-\theta /2$.

## 3 Results

**Fig. 1:**
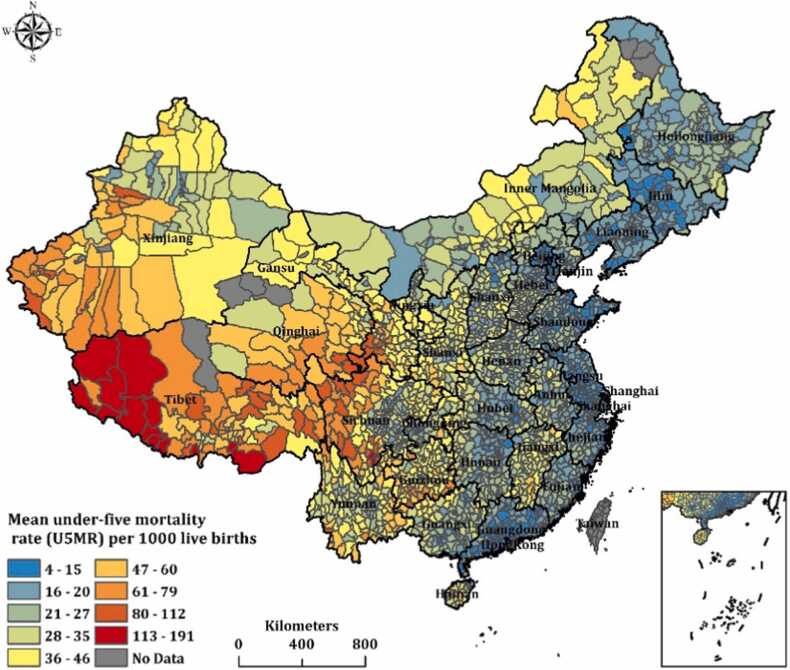
Mean U5MR distribution of 2851 counties in China from1999 to 2012.

The spatial distribution of U5MR in 2581 counties in China over 1999–2012 is shown in Fig. 1. The Southwest region experienced the highest U5MR, with a rate between 113 and 191 per 1000 live births in west Tibet, followed by the Northwest region (including Qinghai, Xinjiang) (47–112 per 1000 live births). In contrast, Eastern and Southcentral regions experienced the lowest U5MR, ranging between 6 and 18 per 1000 live births.

### 3.1 Model selection

We selected the best-fitted model according to DIC, WAIC, MSE, and MAE values, and model complexity. Table S1 shows the above-mentioned values for each model and model performance to explore the impact of PM_2.5_ on U5MR. We found that compared with Model 1 and 2, the Bayesian Model 3 had the lowest DIC, WAIC, MSE, and MAE values of 163109, 157361, 0.201483, and 0.093641, respectively. These results indicated that the spatiotemporal interaction model was the best-fitted model. Furthermore, Model 1 exhibited a negative mean value for PM_2.5_, indicating that the efficiency of the classic parameter model to investigate the impact of ambient PM_2.5_ on U5MR was poor. However, the spatially varying non-parametric and spatiotemporal interaction models were well-fitted for investigating the effect of PM_2.5_ on U5MR with positive mean values. We also calculated the Variance Inflation Factor (VIF) between explanatory variables to detect multicollinearity and to ensure the model is properly specified and functioning correctly. The variance estimated coefficient values of PM_2.5_, GDP, NDVI, temperature, and NTL were 1.29, 1.47, 2.09, 1.95, and 1.04, respectively, suggesting that input variables were not strongly correlated with each other (all VIF < 5).

### 3.2 Spatiotemporal trend of U5MR

**Fig. 2:**
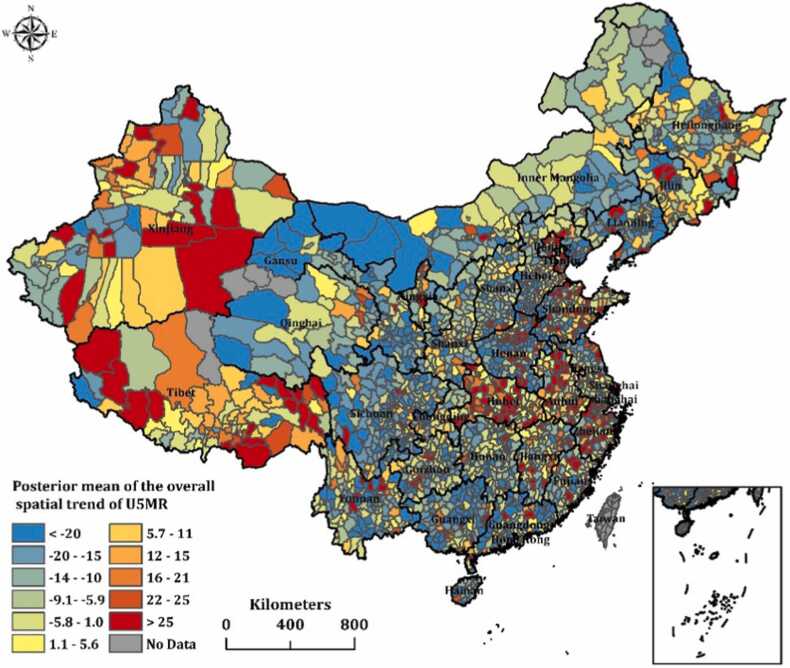
The overall spatial trend distribution of U5MR ${(\xi }_{i})$ in 2851 counties in China from 1999 to 2012.

Fig. 2 shows the spatial distribution of the convolution parameters $\xi_{i}$ posterior mean of the U5MR over the study period, the U5MR appeared to be randomly dispersed with no explicit pattern (unstructured heterogeneity), indicating an outweigh of spatially unstructured residual effects over spatially structured effects. Counties that exhibited high U5MR were primarily located in Xinjiang, Tibet, Hubei, Anhui, and Zhejiang. By comparison, counties with low U5MR were mostly located in Gansu, Inner Mongolia, Qinghai, Sichuan, and Guangxi.

**Fig. 3:**
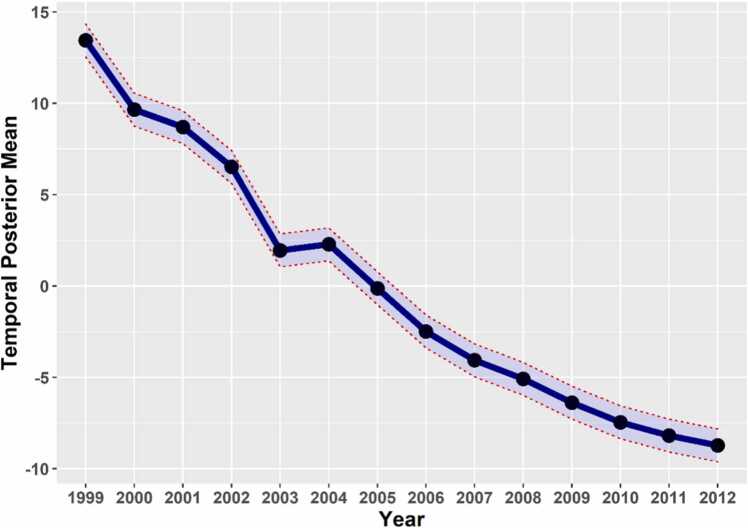
The overall temporal trend of U5MR in 2851 counties, 1999–2012.

The overall temporal trend, ${\omega (\gamma }_{t}+\varphi_{t}￼$) exhibited a downward trend over the period of 1999–2012 (Fig. 3).

For the posterior mean distribution of the $\delta_{\mathrm{it}}\;$of the U5MR for the 14 years of 1999–2012, Fig. 4 shows U5MR space-time interaction distribution, counties with high values from 1999 to 2004, were mainly located in Southwest and Northeast regions, with different spatial patterns by year. Spatial and temporal downward trends were observed after the year 2005.

**Fig. 4:**
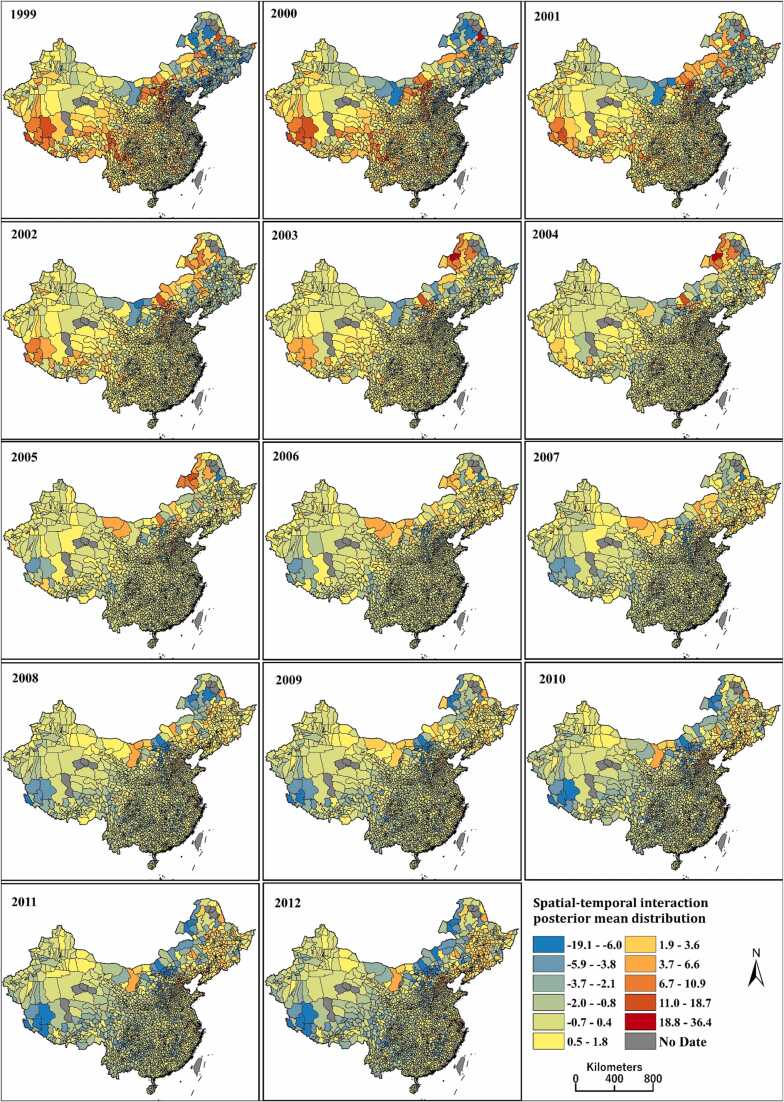
Spatiotemporal distribution of U5MR in 2851 counties over the period 1999–2012.

### 3.3 Associations between PM_2.5_ and U5MR

We estimated the influence of PM_2.5_ on U5MR, first at the national level including all 2851 counties, then at the regional level by separating the counties into six regions. The estimated results from both levels are shown in Table 1. Nationally, PM_2.5_ was significantly and positively associated with U5MR; when PM_2.5_ increased 10 µg/m^3^, U5MR increased by 1.2 (95% confidence interval 1.0 – 1.3) per 1000 live births. However, in subnational regions, such patterns were more evident in Northeastern China with a significant mean value of 1.8 (95% confidence interval 1.4–2.1), followed by Northern China with a mean value of 0.7 (95% confidence interval 0.5–0.9), and Southwestern China (although the positive association was not statistically significant).

**Table 1:** The posterior mean of PM_2.5_ at the national and subnational levels with standard deviation and 95% CL.

Region	Mean	SD	Confidence Interval 25% 95%	
Nationwide	0.117	0.006	0.105	0.129
East China	-0.018	0.009	-0.036	0.001
North China	0.074	0.012	0.051	0.097
Northeast China	0.182	0.017	0.148	0.215
Northwest China	-0.042	0.042	-0.125	0.039
Southcentral China	-0.023	0.010	-0.043	-0.002
Southwest China	0.039	0.030	-0.020	0.098

### 3.4 Sensitivity analysis

**Fig. 5:**
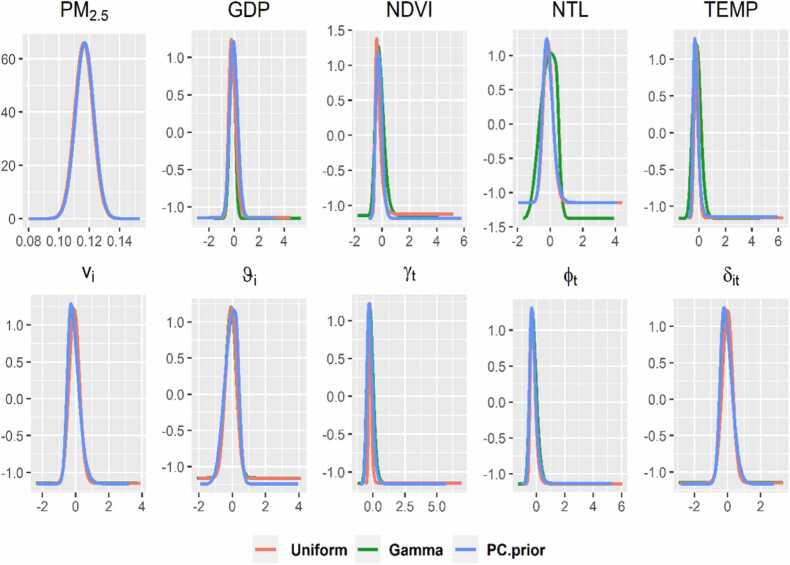
Sensitivity analysis on the priors precisions of the spatiotemporal interaction model. *Note: The hyperparameters*$v_{i},\vartheta_{i}\;$*refer to spatial unstructured residual, and structured residual.*$\gamma_{t}$*,*$\varphi_{t}$*refer to temporal unstructured and structured hyperparameters.*$\delta_{\mathrm{it}}$*refers to spatiotemporal interaction hyperparameter*.

We performed sensitivity analyses to investigate our model sensitivity. Fig. 5 shows the coefficient posterior distribution of the covariate PM_2.5_ and of the hyperparameters $\left\{\tau \nu ,\tau \upsilon ,\;\tau \gamma ,\tau \varphi ,\tau \delta ,{\tau \beta }_{\mathrm{GDP}},\ldots ,{\tau \beta }_{\mathrm{TEMP}}\right\}$ assuming the mentioned above priors. No dramatic change is observed, which indicates that the model is robust.

### 3.5 Embedding dimension, nonlinearity test

We used simplex projection to calculate the embedding dimension for the response variable, U5MR, and its driver, ambient PM_2.5_. As illustrated in Fig. S2, the black dashed line defines the optimal embedding dimension E, with the highest correlation coefficient ρ value (blue line), and lowest MAE (red line). The embedding dimension values for U5MR and PM_2.5_ were 7 and 5, respectively. To identify nonlinearity, we implemented the s-map EDM function. If the parameter θ > 0, the variables have a nonlinear system. However, if θ = 0, the variables have a linear system. As shown in Fig. S3, the θ values (dash line) for U5MR and PM_2.5_ were 6 and 4, respectively, demonstrating a clear nonlinear state dependence, which is crucial for better prediction than linear systems. Cross mapping was computed with a library size of 800 time-series data points and 300 random generated samples for each library, generating best embedding dimension E = 7 and 5.

### 3.6 Potential causal association between PM_2.5_ and U5MR

**Fig. 6:**
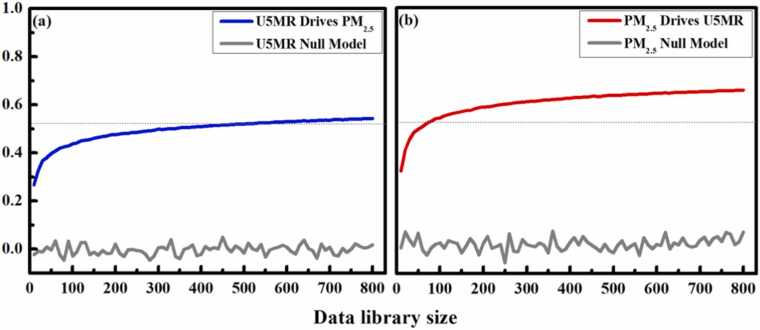
Convergence cross mapping (CCM).

Fig. 6 shows the cross-mapping results between U5MR (blue line) (a) nonsensical direction of causality, and PM_2.5_ (red line) (b). Clear evidence of convergence for PM_2.5_ cross-mapping U5MR was observed, which suggests that U5MR was causally forced by PM_2.5_. The cross-mapping skill of the Seasonal and Ebisuzaki null models (gray line) was relatively low with estimated *p* < 0.05, in comparison with original data CCM max influence value. This implies that causality between PM_2.5_ and U5MR was beyond seasonality, rejecting the null hypothesis that the causality was forced due to synchronized period fluctuation (seasonality). The dashed line in Fig. 6 describes the linear correlation value between U5MR and PM_2.5_.

## 4 Discussion

In this study, we used Bayesian inference spatiotemporal models to estimate spatial and temporal trends of U5MR in China during 1999–2012, and further examined the association between ambient PM_2.5_ and U5MR. Our study revealed that ambient PM_2.5_ exposure was significantly associated with a higher mortality rate among children under the age of five. Our further subnational analysis observed geographical differences across the country with the strongest associations in Northeast China.

We attempted to investigate whether there is a casual relationship between PM_2.5_ exposure and U5MR using an empirical dynamic modeling approach. The analysis suggested that there might be a causal relationship between ambient PM_2.5_ exposure and U5MR, which is badly needed and rarely investigated in previous studies. The relationship between two variables may be spurious. Thus, when investigating their causal relationship, it is important to highlight that the association among these variables are not affected by other confounding variables.

Previous studies have reported significant associations between atmospheric temperature, NDVI, and economic status with childhood mortality (Xu et al., 2012, Shively et al., 2015, Pérez-Moreno et al., 2016). Therefore, we included these variables as important covariates in our analysis. Both GDP and night-time light have previously served as indicators of economic conditions and human activity (Mellander et al., 2015). We included these two variables as random effect variables, though they may be linearly related. We then calculated the Variance Inflation Factor (VIF) and did not find significant collinearity.

Our findings are in line with several studies which also reported a statistical association between ambient fine particulate matter and mortality in children under five years. For example, one study by Egondi et al. indicated that exposure to elevated levels of PM_2.5_ was associated with a high mortality rate among children (Egondi et al., 2018). Another study by Anwar et al. investigated the relationship between PM_2.5_ and child mortality using the least square method, and found that a one unit increase in annual PM_2.5_ concentration was associated with a 14.5% increase in U5MR (Anwar et al., 2021). In the present study, we applied a Bayesian inference approach, which takes prior parameter information into account, and as such is a more flexible method to illuminate the relationship (Elster and Wübbeler, 2015).

After particulate matter is inhaled, the particles could result in cardiovascular and respiratory illness. For children, high levels of PM_2.5_ exposure could lead to acute low respiratory infections include pneumonia which is one of the determinant cause of mortality among children (Liu et al., 2017, Ruan, 2021, Shi et al., 2021). One study found that each 10 μg/m^3^ increase in PM_2.5_ could result in an increase in pneumonia hospitalization by 1.21% (Wang et al., 2021).

In 2012, there were a total of 2857 county-level administrative unites in Mainland China according to the National Bureau of Statistics of China. Among them, 2851 were included in our analysis according to data availability of under-five mortality rates. The 2851 counties were divided into six regional areas, according to the administrative distribution in China. The purpose of dividing the overall study area into separate regions was to more accurately capture the impact of PM_2.5_ on U5MR that may vary by population composition. Our results suggested that PM_2.5_ is indeed a potential risk factor for U5MR, nationally. In subnational analysis, we observed varying patterns of effects of PM_2.5_ among different regions, with stronger effects observed in the North and Northeast regions. By contrast, negative associations between PM_2.5_ and U5MR were found in the Southcentral region. Interestingly, the strength of the association of PM_2.5_ in the Northeast and North regions was greater than the other four regions. This indicates that efforts aimed to control air pollution should be strongly reinforced throughout the entire country, with more urgent needs in Northeast and North China regions. The differential associations between PM_2.5_ and U5MR in different regions are likely to be attributable to the different unknown or unaccounted for environmental and socioeconomic confounding variables; and the concentration of PM_2.5_ chemical composition in each region, such as (sulfate, ammonium, and sodium ion) (Dominici et al., 2015). In addition to, population susceptibility and vulnerability to air pollution, with greater and lower exposure risk in some population subgroups than the general population (Fann et al., 2018).

In this study, we used the INLA method to compute the mean posterior probability of PM_2.5_ parameters to evaluate the effect of PM_2.5_ on U5MR. Specifically, we introduced risk factors as fixed effect variables, including one variable at a time with different latent models’ specifications, while recording DIC and WAIC values at each process. Spatiotemporal regression models 1–3 have the lowest DIC and WAIC values. We also used the empirical dynamic model (EDM), which identified the causal factors of a response variable in a nonlinear dynamic system, which has been widely used in ecological and epidemiological studies (Wu et al., 2020).

The findings from this study possess important public health implications. To improve overall health of children, we suggest that more stringent air pollution control measures should be adopted. Children are more vulnerable to the effects of air pollution due to their immature lungs and developing immune systems, and they may suffer long term impairment as a result of early-stage exposure to particulate matter (Buka et al., 2006). Therefore, our findings support the need for the implementation of effective environmental protection and air pollution control measures throughout China.

Several limitations should be noted. First, more recent data on U5MR (after 2012) was not available. Further investigations based on more recent and updated data are needed. Second, this analysis was based on county-level data and therefore could be subject to ecological fallacy. Third, though we controlled for a series of potential confounding factors in our analysis, some unknown and unavailable potential confounding factors, such as nitrogen dioxide and ozone, might have affected the observed association between PM_2.5_ and U5MR and may have led to residual confounding.

## 5 Conclusion

China exhibited a downward trend in U5MR from 1999–2012 with spatial heterogeneity observed randomly over 31 Chinese provinces across the country. We further provide evidence that higher levels of ambient PM_2.5_ could lead to higher U5MR, and our empirical dynamic model indicates that this association might be causal.

## Funding

This study was supported by the 10.13039/100000865Bill & Melinda Gates Foundation [Grant Number: INV-016826] and one project of Guangdong Provincial Department of Science and Technology.

## CRediT authorship contribution statement

**Sameh. M. M. Alnwisi:** Conceptualization, Methodology, Data curation, Formal analysis, Validation, Visualization, Writing - original draft. **Chengwei Chai:** Visualization, Writing – review & editing. **Bipin Kumar Acharya:** Visualization, Writing – review & editing. **Shiyu Zhang:** Visualization, Writing – review & editing. **Aaron M. Qian:** Visualization, Writing – review & editing. **Zilong Zhang:** Visualization, Writing – review & editing.**Michael G. Vaughn. PhD:** Visualization, Writing – review & editing. **Hong Xian. PhD:** Conceptualization, Visualization, Writing – review & editing. **Qinzhou Wang:** Conceptualization, Formal analysis, Methodology, Validation, Writing – review & editing, Supervision.**Hualiang Lin:** Conceptualization, Formal analysis, Methodology, Validation, Writing – review & editing, Supervision.

## Declaration of Competing Interest

The authors declare that they have no known competing financial interests or personal relationships that could have appeared to influence the work reported in this paper.
