# Supplementary material for: Empirical dynamic modeling of the association between ambient PM2.5 and under-five mortality across 2851 counties in Mainland China, 1999–2012
Source: Ecotoxicol Environ Saf. 2022 Jun 1;237:113513. doi: 10.1016/j.ecoenv.2022.113513 (PMC9061697; doi:10.1016/j.ecoenv.2022.113513)
Supplement: Supplementary file 1 — Supplementary material. [file mmc1.docx]

**Supplementary Materials**

**
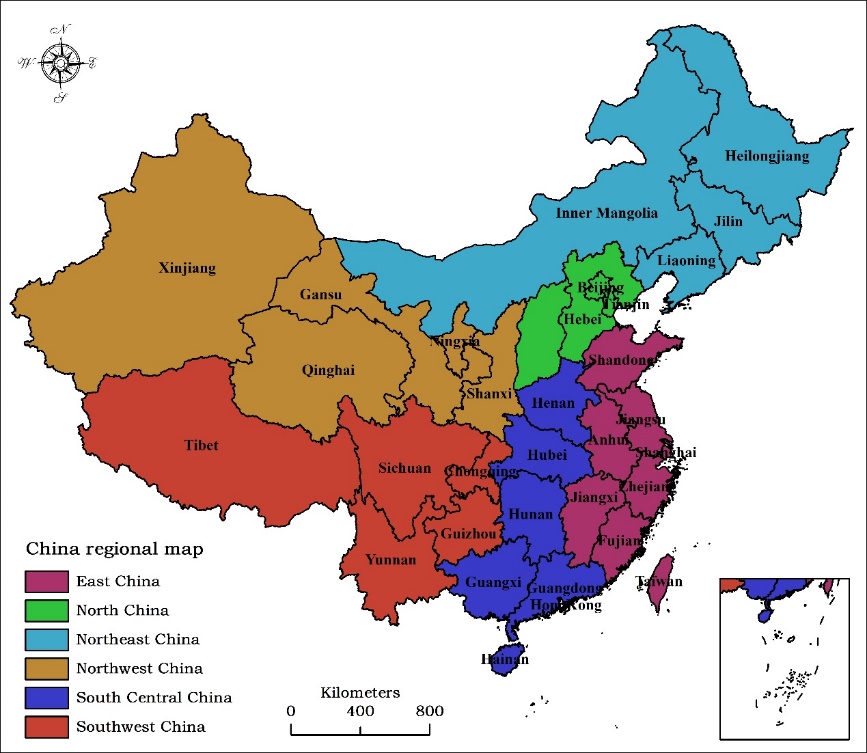
**

***Figure S1****. China classified regional map.*

| Model | Mean | Confidence Interval  25% 95% | DIC | WAIC | MAE | MSE |
| --- | --- | --- | --- | --- | --- | --- |
| Classic | **-0.091** | **-0.097 -0.085** | **201649** | **197737** | **0.85967** | **1.97693** |
| Non-parametric | **0.114** | **0.103 0.126** | **244713** | **242878** | **1.55692** | **5.88603** |
| Space-time Interaction | **0.117** | **0.105 0.129** | **163109** | **157361** | **0.20148** | **0.09364** |

***Table (S1):*** *Statistics summary of the three applied INLA models. (Mean) indicates the mean posterior distribution value of PM_2.5_, Deviance information criterion (DIC), Watanabe-Akaike information criterion (WAIC), Mean absolute error (MAE), and Mean square error (MSE).*


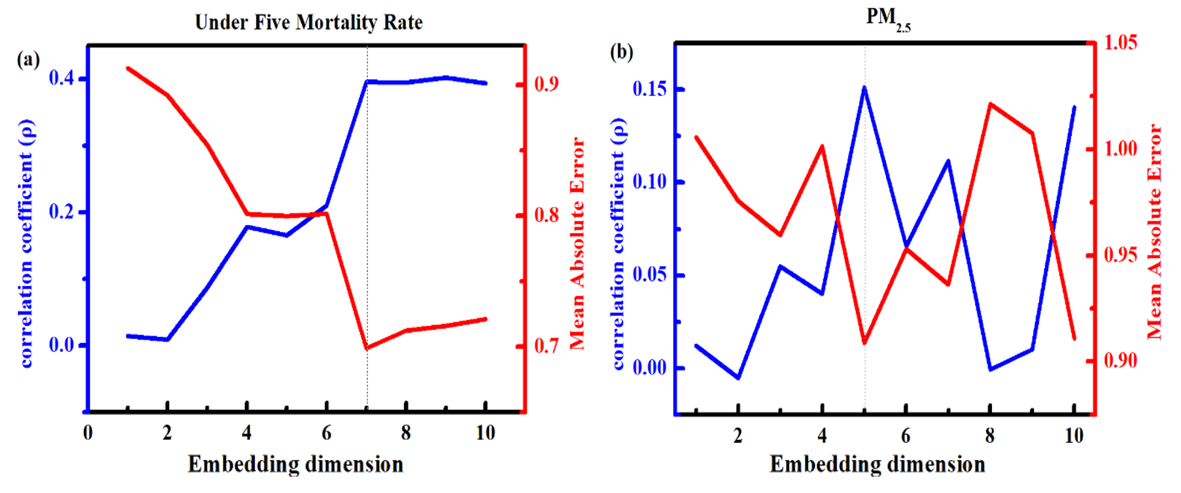


***Figure S2.*** *Optimal embedding dimension E*


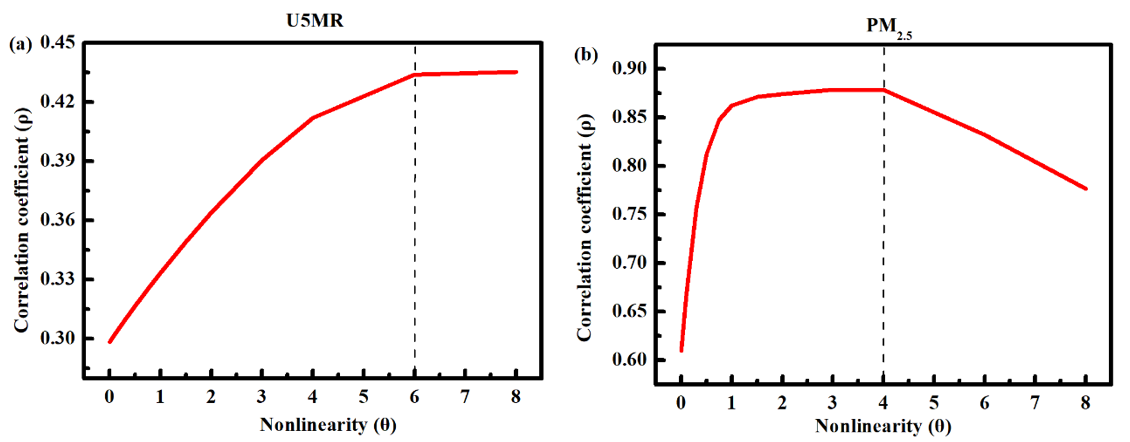


***Figure S3.*** *S-map nonlinearity test.*
